# Triple-channel microreactor for biphasic gas–liquid reactions: Photosensitized oxygenations

**DOI:** 10.3762/bjoc.7.134

**Published:** 2011-08-24

**Authors:** Ram Awatar Maurya, Chan Pil Park, Dong-Pyo Kim

**Affiliations:** 1 National Creative Research Center of Applied Microfluidic Chemistry, Chungnam National University, Daejeon, 305-764, South Korea, www.camc.re.kr; 2 Graduate School of Analytical Science and Technology, Chungnam National University, Daejeon, 305-764, South Korea, Fax: (+82)-42-823-6665

**Keywords:** gas–liquid reaction, microreactor, photosensitization, singlet oxygen

## Abstract

A triple-channel microreactor fabricated by means of a soft-lithography technique was devised for efficient biphasic gas–liquid reactions. The excellent performance of the microreactor was demonstrated by carrying out photosensitized oxygenations of α-terpinene, citronellol, and allyl alcohols.

## Introduction

Microreactors have recently attracted much interest among the scientific community for performing laboratory operations on small scales [1–23]. One of the major driving forces for the development of these devices is their unique characteristics compared to those of classical reaction vessels, such as large surface-to-volume ratio, diffusion dominated mass transfer, fast and efficient heat dissipation, and the capability of spatial and temporal control of the reagents or products. These advantages have been exploited for various purposes such as performing selective reactions with highly unstable intermediates [24–25], improving heterogeneous catalysis [26–29], multi-step synthesis [30–31], process safety [32–34], photo-reactions [35–39], gas-liquid reactions [40–43], etc.

**Figure 1:**
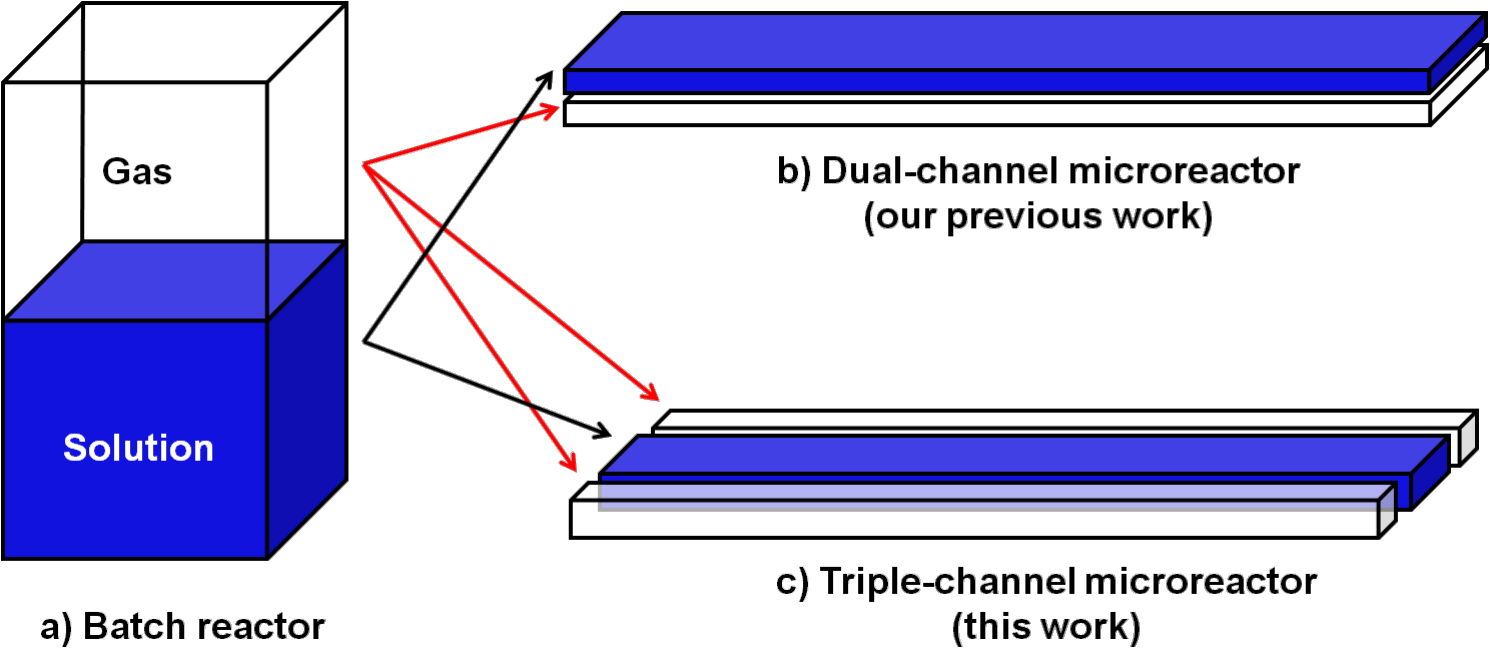
Schematic the for contacting modes of biphasic gas–liquid in (a) batch reactor, (b) dual-channel, and (c) triple-channel microreactors.

In a biphasic gas–liquid reaction, mass transfer from the gas phase to the liquid phase proceeds through the interfacial area. In traditional batch reactors, the interfacial area between the gas and liquid phases is quite small and the ratio of the interfacial area to the volume further decreases with the volume of the reaction mixture. Thus, in scale-up batch reactors the rate of reaction is significantly decreased due to the considerably reduced interfacial-area-to-volume ratio. Therefore, vigorous stirring, ultrasonic agitation, high pressure or supercritical conditions are typically applied to enhance mass transfer in batch reactors for gas–liquid biphasic reactions. Recently, we reported a dual-channel microreactor that dramatically improved the reaction rate of biphasic gas–liquid reaction by enhancing the effective interfacial area [44–45]. In the dual-channel microreactor, in which the top and bottom channels were separated by a thin polydimethylsiloxane (PDMS) membrane, gas from the bottom channel diffused into the solution of the top channel. Thus, only one face of the solution channel was exposed to the gas. Herein, we present an advanced version of the dual-channel microreactor in the form of a triple-channel microreactor where the reaction channel is exposed to gas from two sides, which further increases the effective interfacial area (Figure 1). More importantly, the process of fabrication of the triple-channel microreactor is simpler than for the dual-channel.

## Results and Discussion

**Figure 2:**
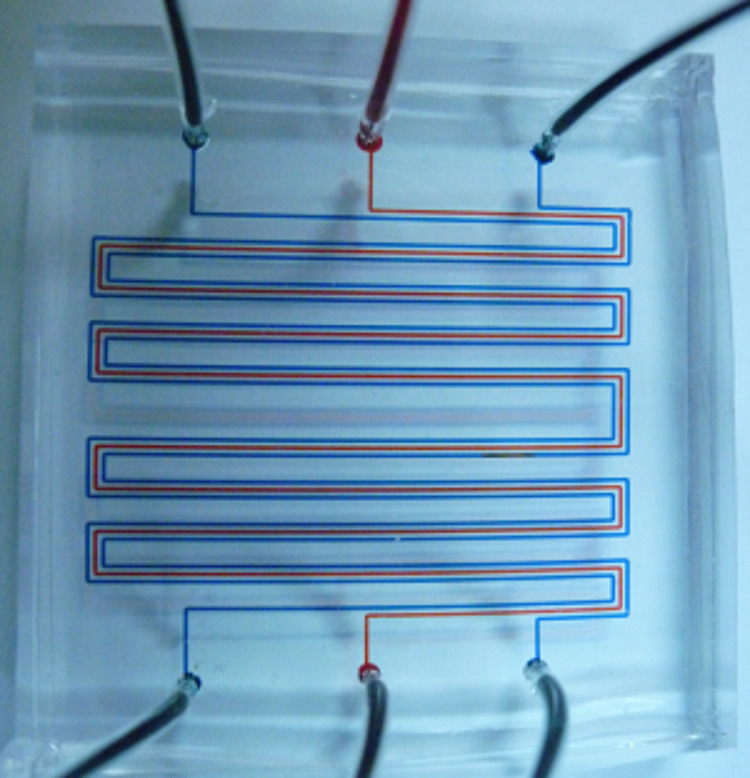
Optical image of the triple-channel microreactor (for demonstration purposes, the inner channel for reaction is filled with red solution and outer channels for gas with blue solution).

PDMS was used to fabricate the triple-channel microreactor for biphasic gas–liquid reactions. It is the most commonly used material for microfluidic devices due to the ease of fabrication and its optical transparency. Although microfluidic devices made of PDMS suffered from swelling problems caused by nonpolar organic solvents [46], we found that organic reactions can conveniently be performed in polar organic solvents such as DMF, DMSO, acetonitrile, etc., without any noticeable problems. The dimension of the middle channel of the fabricated triple-channel microreactor was 33 cm × 250 μm × 40 μm (volume = 3.3 μL). The outer parallel channels were 250 μm in width and 40 μm in depth. The membrane separating the parallel channels was 100 μm thick (for details of the fabrication, see the Supporting Information File 1); an optical image of the fabricated microreactor is shown in Figure 2.

Photosensitized oxygenation was chosen as a biphasic gas–liquid reaction to study the efficiency of the triple-channel microreactor. Photooxygenations in classical reaction vessels suffer from long reaction times due to restricted spatial illumination. In addition, the short lifetime of singlet oxygen in solutions, the low interfacial area between the oxygen and the reaction solution, and the long molecular diffusion distances significantly reduce the reaction efficiency. In this context triple-channel microreactors could be quite useful as they comprise all the required elements for photosensitized oxygenations, namely continuous-flow processing, large gas–liquid interfacial area, short molecular diffusion distances, and very high surface illumination homogeneity.

**Figure 3:**
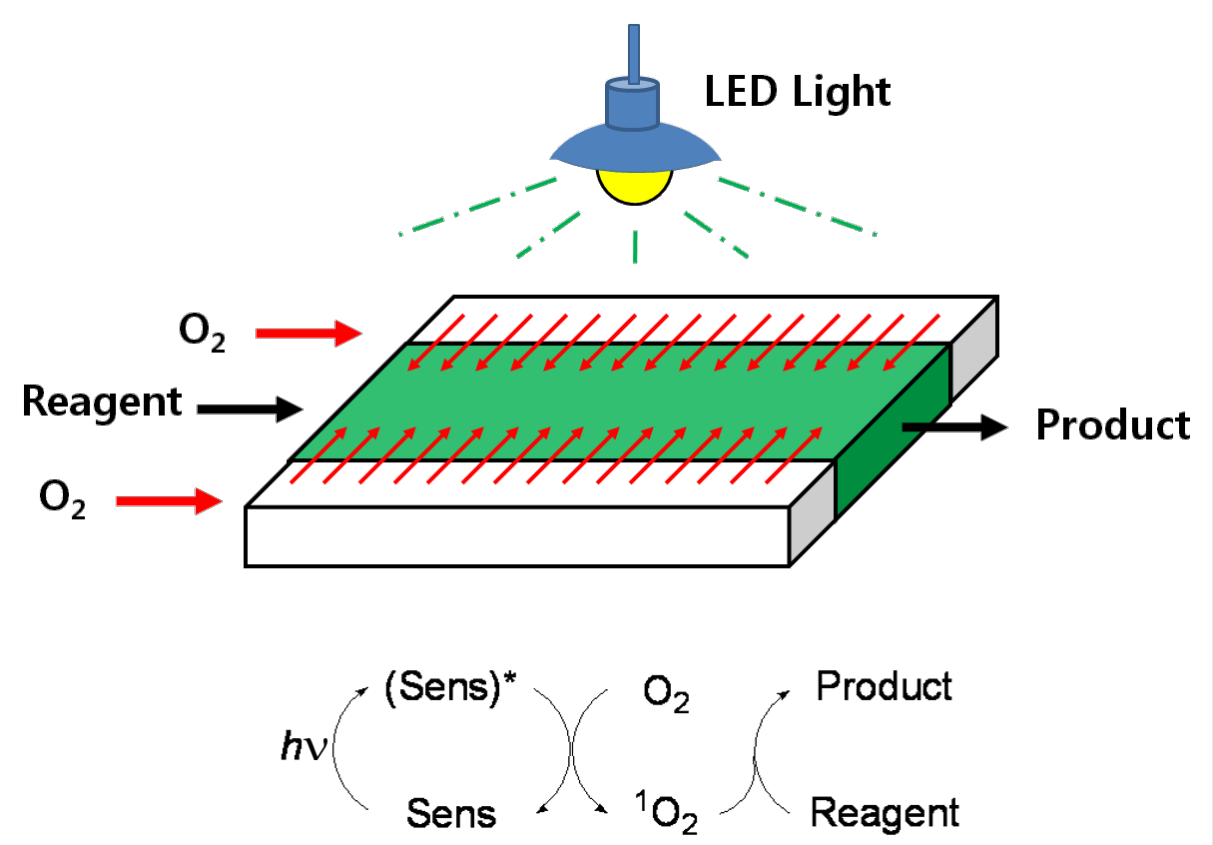
Photosensitized oxygenation in the triple-channel microreactor.

The middle channel was used for the flow of the reaction solution containing the reactant and a photosensitizer, whereas the outer two channels were used for oxygen supply (Figure 3). Efficient oxygen supply to the reaction mixture was achieved by adjustment of the pressure in the outer channels. The overpressure of oxygen in the outer channels results in the generation of bubbles in the middle channel, which affects the control of reagent flow by disturbing the flow rates. Therefore, the pressure of oxygen in the outer channels should be controlled in order to avoid bubble generation in the middle channel and to prevent diffusion of solvents from the middle channel to the outer channels. Thus, only oxygen diffuses into the solution and not the opposite way around. The efficiency of the triple-channel microreactor was studied by carrying out photosensitized oxygenation of citronellol, allyl alcohols, and α-terpinene.

### Photosensitized oxygenation of (−)-citronellol

**Scheme 1:**
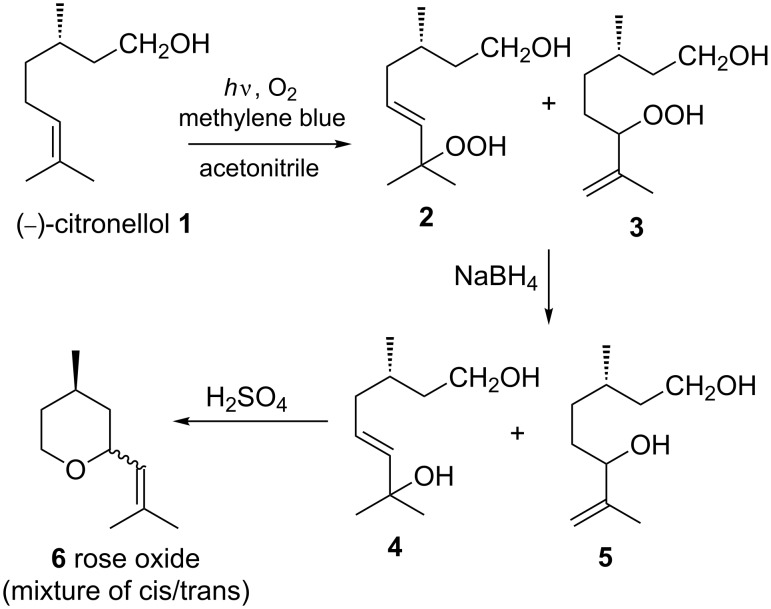
Photosensitized oxygenation of citronellol (a key step in the synthesis of rose oxide).

The photosensitized oxygenation of citronellol is an industrially important synthetic transformation [47] as it is used for bulk production of a fragrance, rose oxide (Scheme 1). The reaction was performed with methylene blue as a sensitizer in acetonitrile.

Table 1 represents the technical data for a typical batch reactor (50 mL round bottom flask), dual channel and triple channel microreactors. Since the illuminated area/volume and gas–liquid interfacial area/volume were highest in the case of triple-channel microreactor, much improved results were expected with the new triple-channel microreactor.

**Table 1:** Technical data for the batch reactor and triple-channel microreactor.^a^

	batch reactor	dual^b^	triple^c^

volume	50 mL	38.9 μL	3.3 μL
illuminated area	15.2 cm^2^	1.98 cm^2^	0.825 cm^2^
illuminated volume	20 mL	38.9 μL	3.3 μL
illuminated area/volume	0.76 cm^−1^	50.9 cm^−1^	250 cm^−1^
gas–liquid interfacial area/volume	0.76 cm^−1^	50.9 cm^−1^	80 cm^−1^

^a^For calculations see Supporting Information File 1. ^b^Dual-channel microreactor, for details of the fabrication and the results of photosensitized oxygenation in the dual-channel microreactor, see reference [45]. ^c^Triple-channel microreactor.

The microreactor was irradiated with a 16 W white LED light source (FAWOO-Tech. Korea, LH16-AFE39S-White) kept in close contact. The reaction mixture (a solution of citronellol and methylene blue in acetonitrile) was pumped into the middle channel without any presaturation with O_2_. The outer channels were closed from one end and oxygen was pumped at a flow rate that was 10 times higher than that of the solution in the middle channel. The batch reaction was performed in a 50 mL round bottom flask and irradiated with the same light source.

In the microreactor the reaction was completed within a few minutes whereas it took several hours to complete in the flask. The results are attributed to very high gas–liquid contact area and illumination area-to-volume ratio for the microreactor compared to that of the round bottom flask. In addition, high illumination homogeniety of the microreactor also plays an important role. These factors make the triple-channel microreactor quite promising for photosensitized oxygenation reactions. The ratio of hydroperoxides **2** and **3** were found to be identical (1:1.5 as determined by ^1^ H NMR) in both the microreactor and the batch reactor. It was particularly noticeable that reactions carried out under higher reactant concentration in the triple-channel microreactor took almost the same time to reach completion as at lower concentration, whereas in the batch reaction conversion was incomplete even after several hours (Table 2).

**Table 2:** Photosensitized oxygenation of (−)-citronellol.

entry	conc.	time	conversion (%)^a^		STY (mmol L^−1^ min^−1^)^b^	

			microreactor	batch	microreactor	batch

1	0.1 M	2 min	99	5	49.5	2.5
2	0.1 M	6 h	—	93	—	0.28
3	0.2 M	2 min	99	—	99	—
4	0.3 M	2 min	91	—	136.5	—
5	0.3 M	6 h	—	63	—	0.53

^a^Conversions were determined by ^1^H NMR using an internal standard. ^b^Space–time yield (STY) = mmol of products/(reactor volume × time).

To compare the productivity for scale-up synthesis, the space–time yield of the triple-channel microreactor and that of the round bottom flask was calculated at various times during the course of the reaction. The space–time yield data reveals that triple-channel microreactors are quite promising for performing efficient photosensitized oxygenation of citronellol in condensed solutions that would minimize the waste of solvents.

### Photosensitized oxygenation of allylic alcohols

The photosensitized oxygenation of allylic alcohols was taken as a second model reaction to illustrate the efficiency of the triple-channel microreactor. The product of this reaction is an allyl hydroperoxide alcohol that is used in the synthesis of artemisinin-derived antimalarial 1,2,4-trioxanes [48]. The reaction in the triple-channel and in batch was carried out as aforementioned with methylene blue as sensitizer. The results from the triple-channel microreactor and the batch reaction presented in Table 3 clearly indicate the efficiency of the former.

**Table 3:** Photosensitized oxygenation of allyl alcohols.

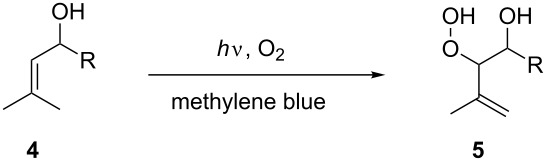							

entry	conc.	time	R	conversion (%)^a^		STY (mmol L^−1^ min^−1^)^b^	

				microreactor	batch	microreactor	batch

1	0.2 M	2 min	H	98	6	49	3
2	0.2 M	2 min	Me^c^	97	—	97	—
3	0.3 M	2 min	H	95	—	142.5	—
4	0.3 M	6 h	H	—	62	—	0.52

^a^Conversions were determined by ^1^H NMR using an internal standard. ^b^Space–time yield (STY) = mmol of products/(Reactor volume × time). ^c^Syn/anti ratio = 75:25 as determined by ^1^H NMR.

### Photosensitized oxygenation of α-terpinene

The photosensitized oxygenation of α-terpinene is a Diels–Alder type [4 + 2] cycloaddition reaction. The product of the reaction is ascaridole, which is widely used as an anthelmintic drug, in tonic drinks and in food flavoring [49]. The reaction was successfully carried out in the triple-channel microreactor with methylene blue as a sensitizer, as above. Significant reduction in the reaction time was again observed when compared to the batch reaction (Table 4). Very high space–time yield of the triple-channel microreactor in comparison to the batch reactor indicates that the microreactor is quite suitable for scaled-up production of the ascaridole.

**Table 4:** Photosensitized oxygenation of α-terpinene.

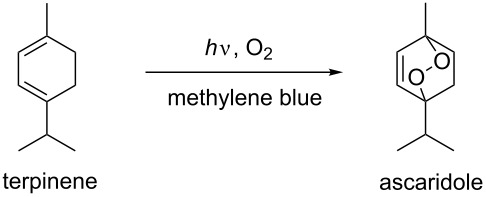						

entry	conc.	time	conversion (%)^a^		STY (mmol L^−1^ min^−1^)^b^	

			microreactor	batch	microreactor	batch

1	0.1 M	1 min	99	7	99	7
2	0.1 M	6 h	—	91	—	0.25
3	0.2 M	1 min	96	—	192	—
4	0.2 M	6 h	—	57	—	0.32

^a^Conversions were determined by ^1^H NMR using an internal standard. ^b^Space time yield (STY) = mmol of products/(Reactor volume × time).

## Conclusion

In conclusion, we developed a triple-channel microreactor for biphasic gas–liquid reactions. In this microreactor, oxygen gas in two outer channels efficiently diffused into the liquid reactants in the middle channel through an extremely large effective interfacial area (area-to-volume ratio). The chemical synthetic efficiency was demonstrated by performing photosensitized oxygenation of α-terpinene, citronellol and allyl alcohols. As a result of the increased illumination as well as the increased gas–liquid contact area per unit volume, the triple-channel microreactor exhibited better performance in the oxygenations of citronellol, allyl alcohols and α-terpinene compared to the batch reactor, or even compared to a typical dual-channel microreactor [44].

## Supporting Information

File 1 Experimental section, analytical data and fabrication of the triple-channel microreactor.
