# Supplementary material for: Triple-channel microreactor for biphasic gas–liquid reactions: Photosensitized oxygenations
Source: Beilstein J Org Chem. 2011 Aug 24;7:1158–63. doi: 10.3762/bjoc.7.134 (PMC3170200; doi:10.3762/bjoc.7.134)
Supplement: File 1 — Experimental section, analytical data and fabrication of the triple-channel microreactor. [file Beilstein_J_Org_Chem-07-1158-s001.pdf]

Supporting Information

for

# Triple-channel microreactor for biphasic gas–liquid reactions: Photosensitized oxygenations

Ram Awatar Maurya,[1] Chan Pil Park,[1] and Dong-Pyo Kim[*1,2]

Address: [1]National Creative Research Center of Applied Microfluidic Chemistry, Chungnam National University, Daejeon, 305-764, South Korea and [2]Graduate School of Analytical Science and Technology, Chungnam National University, Daejeon, 305-764, South Korea, Fax: (+82)-42-823-6665

Email: Ram Awatar Maurya - ram.cdri@gmail.com

Chan Pil Park - chan2seul@hanmail.net

Dong-Pyo Kim - dpkim@cnu.ac.kr, Homepage: www.camc.re.kr

* Corresponding author

## Experimental section, analytical data and fabrication of the triple-channel microreactor

**General:** SU-8-50 photoresist and SU-8 developer were purchased from MicroChem (Newton, MA, USA), while Sylgard 184 silicone elastomer (dimethyl siloxane oligomer) and curing agent (dimethyl, methylhydrogen siloxane, crosslinking agent) were purchased from Dow Corning (Midland, MI, USA). Unless otherwise mentioned, all chemicals were purchased from Aldrich and/or Alfa Aesar and used without further purification. Common organic solvents were purchased from Daejung Chemicals. $^{1}$H NMR and $^{13}$C NMR spectra were recorded on a JNM-AL 400. A white 16 W LED (FAWOO, LH16-AFE39S, emission spectrum range 440-630 nm) was purchased from Lamp-Korea, and was used as the light source in all reactions.

**Fabrication of the triple-channel microreactor:** The parallel triple-channel microreactor was made in two steps as follows:

(a) Fabrication of the silicon master: A 3" silicon wafer was spin coated with SU-8-50 at 500 rpm for 5 seconds and then at 3000 rpm for 30 seconds to get an average thickness of 40 μm. The spin-coated silicon wafer was kept in an oven at 80 °C for 5 minutes and then at 110 °C for 15 minutes for soft baking. A photo-mask (Figure S1) was held over the soft baked silicon wafer and then irradiated with ultraviolet light for 10 minutes. The UV treated silicon wafer was postbaked at 80 °C for 1 minute and then at 110 °C for 4 minutes. The postbaked silicon wafer was kept in SU-8 developer for 5 minute, washed with isopropyl alcohol and dried by air blowing.

(b) Fabrication of PDMS-based triple-channel microreactor: Sylgard 184 from Dow Corning and its curing agent were mixed in 10:1 ratio. After degassing, the mixture was poured over the silicon master and kept in an oven at 60 °C for 3 hours. The cured PDMS mold was peeled off and punctured with a needle to make the tubing connections. The PDMS stamp and a simple PDMS block were bonded together by $O_2$ plasma and then kept in an oven at 100 °C overnight to strengthen the bonding.

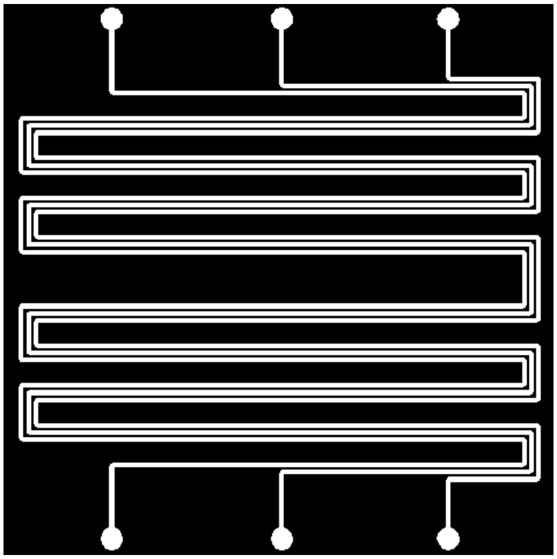

**Figure S1**: Photo-mask used to make the channel.

**Calculation of technical data for the batch reactor and triple-channel microreactor:**

**For batch reaction (50 mL round bottom flask):** For simplicity of the calculations, we assume the condition of no stirring and no bubbling.

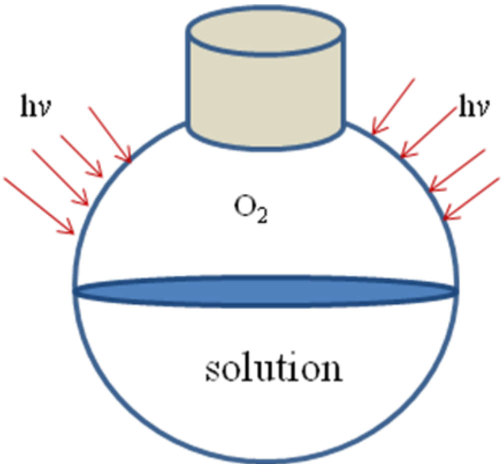


Volume for solution = 20 mL

Illuminated surface area (blue surface) = Oxygen–liquid interface area (blue surface) = $\pi \times (2.2\ \text{cm})^2 = 15.20\ \text{cm}^2$

Contact-area-to-volume ratio = $(15.20\ \text{cm}^2)/20\ \text{mL} = 0.76\ \text{cm}^{-1}$

**For triple-channel microreactor:**

Length of the middle channel (l) = 33 cm

Width of the middle channel (w) = 250 μm

Depth of the middle channel (d) = 40 μm

Volume of the middle channel = lwd = 3.3 μL

Illuminated surface area = lw = 33 cm × 250 x $10^{-4}$ cm

Oxygen-liquid interface area = 2ld = 2 × 33 cm × 40 × $10^{-4}$ cm

**Photosensitized oxygenation of (−)-citronellol in the batch system (general procedure in the batch reaction for photosensitized oxygenation)**: A solution of (−)-citronellol (2 mmol) and methylene blue (0.02 mmol) in acetonitrile (20 mL) was loaded into a 50 mL round bottom flask and was treated with oxygen bubbling for 20 min. The flask was then irradiated with a 16W LED lamp kept in close contact. The ratio of the mixture of products was analyzed by means of $^1$H NMR using an internal standard after $NaBH_4$ treatment of the crude mixture in $CH_3OH$ (Scheme 1S).

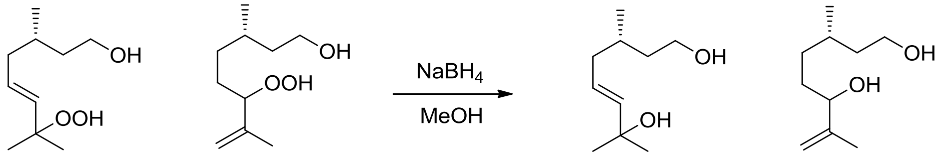


2 3 2S 3S

1 : 1.5

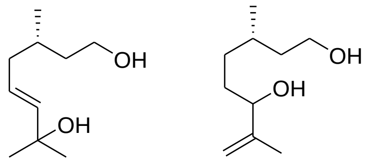


2S 3S

**Scheme S1:**

$^1$H NMR ($CDCl_3$, 400 MHz) δ 8.20 (bs, 1H, OH), 7.90 (bs, 1H, OH), 5.73 (dt, *J* = 15.85, 7.07 Hz, 1H, $CH_2CH$=CH of **2S**), 5.57 (d, *J* = 15.85 Hz, 1H, $CH_2CH$=CH of **2S**), 5.12–5.01 (m, 2H =$CH_2$ of **3S**), 4.31–4.20 (m, 1H, CHOH of **3S**), 3.75–3.63 (m, 4H, $CH_2OH$ of **2S** and **3S**), 2.09–1.93 (m, 2H, $CH_2CHOH$ of **3S**), 1.73–1.25 (m, 12H, $CH_2CHCH_2$ and OH of **2S** and **3S**), 1.60 (s, 3H, $CH_3C$= of **3S**), 1.33 (s, 6H, $(CH_3)_2C$ of **2S** ), 0.93–0.90 (m, 6H $CH_3CH$ of **2S** and **3S**).

**Photosensitized oxygenation of (−)-citronellol in triple-channel microreactors**: A solution of (−)-citronellol (3.0 mmol) and methylene blue (0.03 mmol) in acetonitrile (10 mL) was loaded into a gas-tight Hamilton syringe and delivered into the middle channel while oxygen was delivered into the parallel side channels of the microreactor with irradiation from a 16W LED lamp. The ratio of the mixture of products was analyzed by means of $^1$H NMR after $NaBH_4$ treatment of the crude mixture in $CH_3OH$.

**Photosensitized oxygenation of α-terpinene to ascaridole in the batch system**: The reaction procedure followed the general procedure in batch reaction for photosensitized oxygenation. The conversion was analyzed by means of $^1$H NMR with an internal standard.

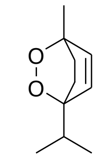


$^1$H NMR ($CDCl_3$, 400 MHz) δ 6.49 (d, *J* = 8.0 Hz, 1H, C$\underline{H}$), 6.41 (d, *J* = 8.0 Hz, 1H, C$\underline{H}$), 2.03–2.06 (m, 2H, $CH_2C\underline{H_2}$), 1.97 (hept, *J* = 6.0 Hz, 1H, $C\underline{H}(CH_3)_2$), 1.49–1.56 (m, 2H, $C\underline{H_2}CH_2$), 1.37 (s, 3H, $C\underline{H_3}$), 0.99 (d, *J* = 6.0 Hz, 6H, $CH(C\underline{H_3})_2$).

**Photosensitized oxygenation of α-terpinene to ascaridole in the triple-channel microreactor**: The reaction procedure followed the general procedure in the triple-channel microreactor for photosensitized oxygenation. The conversions were analyzed by means of $^1$H NMR spectroscopy with an internal standard.

**Photosensitized oxygenation of allylic alcohols in triple-channel microreactors**: The reaction procedure followed the general procedure in triple-channel microreactor for photosensitized oxygenation. The conversions were analyzed by means of $^1$H NMR spectroscopy with an internal standard.

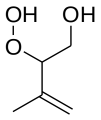


$^1$H NMR ($CDCl_3$, 400 MHz) δ 5.07–5.03 (m, 2H, $C\underline{H_2}C$=), 4.56–4.50 (m, 1H, C$\underline{H}$OOH), 3.76 (d, *J* = 6.58 Hz, 2H, $C\underline{H_2}OH$), 1.78 (s, 3H, $C\underline{H_3}C$=).

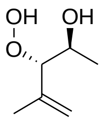


anti

*Anti*: $^1$H NMR ($CDCl_3$, 400 MHz) δ 9.14 (s, 1H, O$\underline{H}$), 5.10–5.03 (m, 2H, $C\underline{H_2}$=), 4.14 (d, *J* = 4.56 Hz, 1H, C$\underline{H}$OOH), 3.89 (dq, *J* = 4.56, 6.48 Hz, 1H, C$\underline{H}$OH), 1.74 (s, 3H, $C\underline{H_3}C$=), 1.14 (d, *J* = 6.45 Hz, 3H, $C\underline{H_3}CH$).
